# Hormonal and Nutritional Features in Contrasting Rootstock-mediated Tomato Growth under Low-phosphorus Nutrition

**DOI:** 10.3389/fpls.2017.00533

**Published:** 2017-04-11

**Authors:** Cristina Martínez-Andújar, Juan M. Ruiz-Lozano, Ian C. Dodd, Alfonso Albacete, Francisco Pérez-Alfocea

**Affiliations:** ^1^Departamento de Nutrición Vegetal, CEBAS-CSICMurcia, Spain; ^2^Departamento de Microbiología del Suelo y Sistemas Simbióticos, Estación Experimental del Zaidín (CSIC)Granada, Spain; ^3^Lancaster Environment Centre, Lancaster UniversityLancaster, UK

**Keywords:** grafting, phytohormones, nutrients, root-to-shoot communication, *Solanum*

## Abstract

Grafting provides a tool aimed to increase low-P stress tolerance of crops, however, little is known about the mechanism (s) by which rootstocks can confer resistance to P deprivation. In this study, 4 contrasting groups of rootstocks from different genetic backgrounds (*Solanum lycopersicum* var. cerasiforme and introgression and recombinant inbred lines derived from the wild relatives *S. pennellii* and *S. pimpinellifolium*) were grafted to a commercial F1 hybrid scion and cultivated under control (1 mM, *c*) and P deficient (0.1 mM, *p*) conditions for 30 days, to analyze rootstocks-mediated traits that impart low (*L*, low shoot dry weight, SDW) or high (*H*, high SDW) vigor. Xylem sap ionic and hormonal anlyses leaf nutritional status suggested that some physiological traits can explain rootstocks impacts on shoot growth. Although xylem P concentration increased with root biomass under both growing conditions, shoot biomass under low-P was explained by neither changes in root growth nor P transport and assimilation. Indeed, decreased root P export only explained the sensitivity of the *HcLp* rootstocks, while leaf P status was similarly affected in all graft combinations. Interestingly, most of the nutrients analyzed in the xylem sap correlated with root biomass under standard fertilization but only Ca was consistently related to shoot biomass under both control and low-P, suggesting an important role for this nutrient in rootstock-mediated vigor. Moreover, foliar Ca, S, and Mn concentrations were (i) specifically correlated with shoot growth under low-P and (ii) positively and negatively associated to the root-to-shoot transport of the cytokinin *trans*-zeatin (*t*-Z) and the ethylene precursor 1-aminocyclopropane-1-carboxylic acid (ACC), respectively. Indeed, those hormones seem to play an antagonistic positive (*t*-Z) and negative (ACC) role in the rootstock-mediated regulation of shoot growth in response to P nutrition. The use of *Hp*-type rootstocks seems to enhance P use efficiency of a commercial scion variety, therefore could potentially be used for increasing yield and agronomic stability under low P availability.

## Introduction

While the low availability of phosphorus in most agricultural soils commonly limits plant growth requiring the application of chemical fertilizers or organic manures, it is considered one of the major contaminants of aquifers following nutrient addition. Moreover, due to the non-renewable nature of rock phosphate reserves, increasing crop phosphorus use efficiency (PUE) is considered a sustainable strategy to reduce potentially catastrophic future P-limitations to agriculture (Davies et al., 2009; Zhang et al., 2014; Cordell and White, 2015). Improving crop P use efficiency could be achieved through conventional/marker assisted breeding and genetic engineering by (i) enhancing P uptake efficiency and/or by (ii) improving P use efficiency. Several studies have identified QTLs and candidate genes that increase P uptake efficiency (mainly related to root architecture and exudates), but few have detected genetic determinants of P use efficiency (Zhang et al., 2014).

Grafting is used as an alternative to breeding in horticultural crops since appropriate and compatible rootstocks can enhance plant performance by improving both nutrient acquisition and utilization efficiency (Lee and Oda, 2010; Gregory et al., 2013; Albacete et al., 2015c; Nawaz et al., 2016). However, although grafting alters concentrations of several macro and micronutrients in the shoot tissues compared to the non-grafted and self-rooted plants (Rouphael et al., 2008), rootstock-mediated physiological effects under nutrient deficit conditions have been rarely studied (Pérez-Alfocea, 2015; Nawaz et al., 2016). For example, *Cucurbita maxima* rootstocks increased leaf P concentration and yield in melon (Ruiz et al., 1997) and watermelon (Colla et al., 2010) compared with self-rooted plants, and increased P uptake and utilization under low P supply. Although rootstocks can improve P uptake and its transport to the leaves, almost no information is available about the underlying rootstock-mediated physiological mechanisms (Albacete et al., 2015c; Pérez-Alfocea, 2015; Nawaz et al., 2016).

An extensive root system is especially important for the uptake of P due to its low mobility in the soil (Marschner and Cakmak, 1986). Low P induces adaptive changes in root system architecture (RSA) that increase soil foraging, such as higher root to shoot ratio, axial roots with shallower growth angles, lateral root proliferation, denser root hairs and the growth of cluster roots (Lambers et al., 2011; Lynch, 2011; Zhang et al., 2014). These are stimulated by local P sensing due to P availability, phytohormone fluxes, sugar signaling, and the availability of other nutrients interactions. P-deficiency can change hormone production, sensitivity and transport to regulate expression of P-responsive genes and RSA (Chiou and Lin, 2011). Plant hormones and other compounds like, sugars, miRNAs and Ca, involved can modulate low-P induced changes to RSA (Shen et al., 2011; Niu et al., 2013). However, while adaptive changes in RSA may increase P uptake efficiency, increases in P use efficiency seem related to systemic signals affecting shoot growth.

Root-shoot communication is essential for plant adaptation to P deprivation (Kapulnik and Koltai, 2016). Thus, P deficiency induces the expression of transporter genes and therelease of organic acids and phosphatase enzymes from roots into the rhizosphere (Vance et al., 2003). However, nutrient sensing by the shoot, along with nutrient assimilation ability and the nutrient assimilation along with the subsequent C partitioning from shoot to root are most severely affected by P deprivation (Cakmak et al., 1994; Hermans et al., 2006). Although underlying mechanisms of P signaling have not been elucidated, P itself, strigolactones, ethylene, cytokinins, and jasmonic acid (JA) may be involved (Chiou and Lin, 2011; Khan et al., 2016). In addition to P-dependent root modifications, ethylene evolutionis regulated by P distribution, and mediates systemic P signaling leading to increases in root/shoot biomass during P deficiency (Nagarajan and Smith, 2012). P deficiency can enhance xylem ABA concentration in response to changing nutrient solution P concentration or soil P availability (Peuke, 2016). Nevertheless, hormones can act both locally and systemically, and it can be problematic to distinguish between these two modes of action (Zhang et al., 2014). In this study, we mainly focus on hormonal changes in the xylem and their systemic role in the nutritional status and growth of the shoot under limited P supply.

Tomato crops require 3.5–4.7 kg ha^-1^ of this nutrient in the field, comprising 0.2–0.3% of the leaf dry matter (Adams, 1986). In semi-hydroponic greenhouse crops, standard P fertilization supplies between 1 and 1.5 mM in the nutrient solution (Cadahia et al., 1995), although this may be lowered to 0.3–0.6 mM depending on phenological status. Below these concentrations, plant growth and yield is reduced and physiological disorders occur (Marschner and Cakmak, 1986). Grafting a unique scion variety onto contrasting rootstocks available in different *Solanum* spp genetic backgrounds offers a new approach to study the role of the root-to-shoot hormonal and nutrient communication in acquiring and using P under suboptimal nutrient availability. This study aims at verifying whether the rootstock could induce variation in shoot vigor under low P conditions by altering root export of P and other nutrients/phytohormones that regulate shoot growth and P use efficiency.

## Materials and Methods

### Plant Material and Growth Conditions

This experiment used a commercial tomato cultivar (*Solanum lycopersicum* L. cv. Boludo F1, Seminis Vegetable Seeds Ibérica S.A., Barcelona, Spain) as scion, which was either self-grafted or grafted onto 144 different rootstocks (Albacete et al., 2015b): a population of 129 recombinant inbred lines (RILs) developed from a salt-sensitive genotype *S. lycopersicum* var. as female parent and a salt-tolerant line from S. *pimpinellifolium* as male parent (Monforte et al., 1997) provided by Instituto Valenciano de Investigaciones Agrarias (IVIA); six accessions derived from a cross between *S. lycopersicum* var. cerasiforme and *S. pimpinellifolium*, selected for drought tolerance (supplied by The World Vegetable Center, AVRDC) and nine introgression lines from *S. lycopersicum* × *S. pennellii* and × *S. habrochaites*, selected for high root/shoot ratio, salinity, and drought tolerances (sourced from The Tomato Genetics Resource Centre, TGRC). Grafting was conducted using the splicing technique at the seedling stage (two to three true leaf) where the scion was attached at the first node of the rootstock (Savvas et al., 2011). One month later, three plants per graft combination were transferred to an experimental greenhouse, randomly distributed and cultivated for a period of 30 days in a semi-hydroponic system using sand as substrate (1.5 kg per pot of 2 l volume and per plant), with 16 h light/8 h dark period at temperatures ranging from 19 to 25°C, a relative 50–60% relative humidity and an average photosynthetic photon flux density of 800 μmol m^-2^ s^-1^, as measured with a light meter (Model LI-188B, LI-Cor, Lincoln, NE, USA). Plants were initially irrigated with a complete Hoagland nutrient solution for 30 days, then half the plants of each graft combination received a reduced P concentration of 0.1 mM (low-P, *p*) compared to the standard P concentration (1 mM, control, *c*) for a period of 30 days. The concentration of the other macro and micronutrients in both standard and modified nutrient solutions were: N, 12.5 mM (NO3:NH4, 12:0.5); K, 6 mM; Ca, 4 mM; Mg, 2 mM; Fe, 100 μM; B, 46 μM; Mn, 9 μM; Zn, 0.76 μM; Cu, 0.75 μM, and Mo 0.02 μM. Each plant received 60 ml of the corresponding Hoagland nutrient solution on alternate days. Three independent experiments were performed with a random distribution of one replicate per graft combination per P concentration.

Thirty days after starting the low-P treatment, the shoot was severed 1.5 cm above the graft union and immediately weighed and oven-dried to determine shoot dry weight (SDW). The second fully expanded mature leaf of 3 plants per graft combination was used to measure leaf P concentration after oven-drying. Xylem sap was obtained by placing the root system in a Scholander-type pressure chamber, applying pressure (0.4 MPa) for 2 min and collecting xylem sap into a pre-weighed Eppendorf tube. Samples were immediately frozen on liquid nitrogen and stored at –80°C until analysis. Sap volume was recorded to calculate the sap flow rate (Netting et al., 2012). After xylem sap collection, the roots were washed from the pot, then oven-dried to determine root dry weight (RDW).

In this study, eight rootstocks were phenotypically selected based on differences in shoot vigor (measured as SDW) and classified into four groups: the first group comprised two rootstocks (RILs 187 and 58) having low vigor (low SDW, *L*) irrespective of P treatment (*LcLp*); the second group was two rootstocks (RILs 130 and 252) showing high vigor (high SDW, *H*)under *c* and low vigor under *p* conditions (*HcLp*); the third group involved two rootstocks (RIL 233 and IL-3-4) having low vigor under *c* and high vigor under *p* conditions (*LcHp*); and the fourth group comprised two rootstocks (TL01749 and TL02254) with high vigor in both treatments (*HcHp*).

### Ion Concentration

Leaves were dried for 48 h at 80°C, milled to power and 200 mg dry tissue was digested with a HNO_3_:HClO (2:1, v/v) solution. Digested leaves and root xylem sap samples were analyzed by using inductively coupled plasma spectrometry (ICP-OES, Thermo ICAP 6000 Series).

### Hormone Analysis

Cytokinins (*trans*-zeatin, *t*-Z, zeatin riboside, ZR, and isopentenyl adenine, iP), the ethylene precursor 1-aminocyclopropane-1-carboxylic acid (ACC), abscisic acid (ABA), jasmonic acid (JA), salicylic acid (SA), and gibberellins (GA_1_, GA_3_, and GA_4_) were analyzed according to Albacete et al. (2008) with some modifications. Briefly, xylem sap samples were filtered through 13 mm diameter Millex filters with 0.22 μm pore size nylon membrane (Millipore, Bedford, MA, USA). Ten microliter of filtrated extract were injected in a U-HPLC-MS system consisting of an Accela Series U-HPLC (ThermoFisher Scientific, Waltham, MA, USA) coupled to an Exactive spectrometer (ThermoFisher Scientific, Waltham, MA, USA) using a heated electrospray ionization (HESI) interface. Mass spectra were obtained using the Xcalibur software version 2.2 (ThermoFisher Scientific, Waltham, MA, USA). For quantification of the plant hormones, calibration curves were constructed for each analyzed component (1, 10, 50, and 100 μg l^-1^).

### Statistics

Correlation analyses and principal component analysis (PCA) were performed using SPSS for Windows (Version 22.0, SPSS Inc., Chicago, IL, USA). Means of different graft combinations and P treatment were compared using Tukey’s test at 0.05 of confidence level and the Varimax method was performed for PCA.

## Results

### Rootstock-mediated Variation in Root/Shoot Growth and Xylem Sap Flow

When the entire tomato population was used as rootstocks, SDW varied by 1.5-fold under low-P supply (0.1 mM) as shown previously (Albacete et al., 2015b). Only 2% of graft combinations had significantly higher SDW than the self-grafted commercial cultivar Boludo F1. Shoot biomass was linearly correlated between control and low-P conditions when the whole population was used as rootstock (*r* = 0.58, *P* ≤ 0.01, data not shown). In the present study, four contrasting groups (2 lines per group) of rootstocks were selected for their differential effect on shoot biomass under control and low-P supply (see Materials and Methods): *LcLp*, *HcLp*, *LcHp*, and *HcHp*.

**FIGURE 1:**
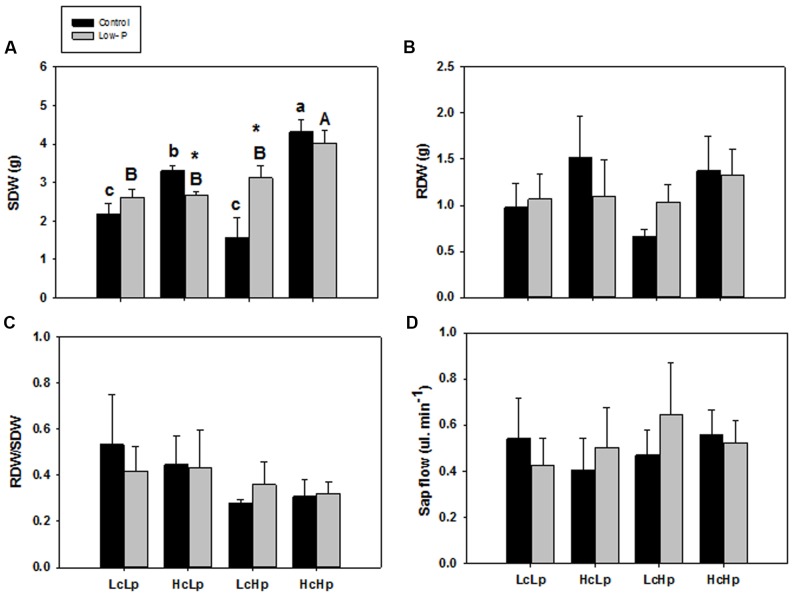
**Shoot dry weight (SDW) (A)**, root dry weight (RDW) **(B)**, RDW/SDW ratio (RDW/SDW) **(C)**, and root sap flow **(D)** of the scion (*Solanum lycopersicum* cv. Boludo F1) grafted onto selected rootstocks for high (H) or low (L) vigor growing under control (c) and low P (p) conditions during 30 days. Different letters indicate significant differences among graft combinations (*n* = 6, *P* < 0.05) within each treatment. ^∗^indicate significant differences between control and low-P treated plants according to the Tukey test (*P* < 0.05).

The *H* rootstocks produced between 1.5 and 2.6 times more SDW than the *L* ones under standard fertilization, while low-P decreased (*HcLp*) or increased (*LcHp*) shoot biomass by 20 and 90% compared to control conditions, respectively (**Figure 1A**). In contrast, root biomass (**Figure 1B**) and the RDW/SDW (**Figure 1C**) were not significantly affected by the rootstock genotype or the P treatment. Indeed, RDW in control and low-P conditions was very closely correlated (*r* = 0.73, *P* ≤ 0.001) in the whole population (data not shown), suggesting little or no adaptive effect in root biomass through low-P supply, as supported by the lack of correlation observed between RDW and SDW under low-P conditions for the selected graft combinations (*r* = 0.25, *P* = 0.267).

There were no significant differences in the xylem sap flow across grafted plants in either treatments (**Figure 1D**), and no relationship was found between this parameter and SDW (**Table 1**). However, a significant negative correlation was found between root xylem sap flow and RDW under standard (*r* = –0.54, *P* ≤ 0.01) and low-P (*r* = –0.46, *P* ≤ 0.05) nutrition (**Table 1**).

**Table 1:** Linear correlation coefficients between shoot dry weight (SDW) and root dry weight (RDW) and ionomic and hormonal related parameters in the leaf and root xylem sap of the scion (*Solanum lycopersicum* cv. Boludo F1) grafted onto selected rootstocks growing under low- P conditions during 30 days.

	Ionic parameters													

	**Macronutrients**						**Micronutrients**						
			**P**	**Ca**	**K**	**Mg**	**Na**	**S**		**Mn**	**B**	**Fe**	**Zn**	
**SDW**	Leaf		–0.097	0.514^∗^	0.064	0.157	–0.106	0.668^∗^		0.809^∗∗^	–0.220	–0.027	0.300	
	Xylem		0.371	0.433^∗^	–0.134	–0.050	–0.369	0.339		0.057	–0.202	0.257	–0.035	
**RDW**	Leaf		0.295	–0.141	0.124	–0.053	0.613^∗^	–0.176		–0.242	0.475	0.276	0.117	
	Xylem		0.463^∗^	0.429^∗^	0.078	–0.155	–0.471^∗^	0.161		0.093	–0.051	0.000	0.042	

	**Hormonal parameters**					

		**SF**	***t***-**Z**	**ZR**	**iP**	**CKs**	**ACC**	**ABA**	**JA**	**SA**	**GA_1_**	**GA_3_**	**GA_4_**	**GAs**

**SDW**	Xylem	0.258	0.505^∗^	–0.087	–0.248	0.498^∗^	–0.491^∗^	0.408	–0.184	–0.339	0.183	0.129	0.003	0.116
**RDW**	Xylem	–0.462^∗^	–0.103	–0.223	–0.594^∗^	–0.116	–0.487^∗^	0.284	–0.310	–0.150	0–0.418	–0.423	–0.661^∗∗^	–0.508^∗^

*Significant correlations (^∗^*P* < 0.05, ^∗∗^*P* < 0.01, n = 12–24) are shown in bold. P, phosphorous; Ca, calcium; K, potassium; Mg, magnesium; Na, sodium; S, sulfur; Mn, manganese; B, boron; Zn, zinc; SF, sap flow; *t*-Z, trans-zeatin; ZR, zeatin riboside; iP, isopentenyladenine, CKs; total cytokinins; ACC, 1-Aminocyclopropane-1-carboxylic acid; ABA, abscisic acid; SA, salicylic acid; JA, jasmonic acid; GA_*1*_, gibberellin A1; GA_*3*_, gibberellin A3; GA_*4*_, gibberellin A4; GAs, total gibberellin*.

### Principal Component Analysis of Rootstock-mediated Response under Low-P

**FIGURE 2:**
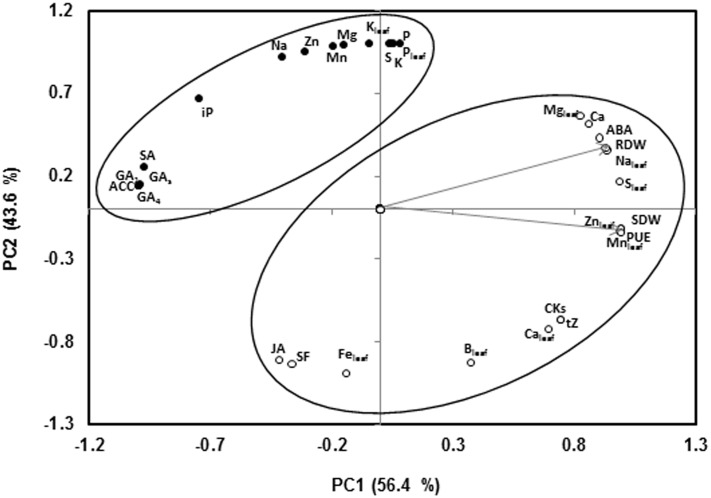
**Two axes of a principal component (PC1, PC2) analysis showing SDW and RDW trait vectors (indicated by arrow) and the position of all variables (denoted by abbreviations) studied under low-P conditions**. Arrows indicate eigenvectors representing the strength (given by the length of the vector) and direction of the trait correlation relative to the first two principal components (PC1, PC2). The circles enclose those variables that fall into the same cluster (75% confidence level). Abbreviations for the variables are given thus: SDW, shoot dry weight; RDW, root dry weight; SF, sap flow; PUE, phosphorous use efficiency; *t*-Z, *trans*-zeatin; iP, isopentenyladenine, CKs; total cytokinins; ACC, 1-Aminocyclopropane-1-carboxylic acid; ABA, abscisic acid; SA, salicylic acid; JA, jasmonic acid; GA_1_, gibberellin A1; GA_4_, gibberellin A4; GAs, total gibberellins; K, potassium in the xylem sap; Na, sodium in the xylem sap; P, phosphorus in the xylem sap; Mg, magnesium in the xylem sap; S, sulfur in the xylem sap; Ca, calcium in the xylem sap, Zn, zinc in the xylem sap; Mn, manganese in the xylem sap; K_leaf_, potassium in the leaf; Na_leaf_, sodium in the leaf; P_leaf_, phosphorous in the leaf; Mg_leaf_, magnesium in the leaf; S_leaf_, sulfur in the leaf; Ca_leaf_, calcium in the leaf; Zn_leaf_, zinc in the leaf; Mnleaf, manganese in the leaf; Fe_leaf_, iron in the leaf and B_leaf_; boron in the leaf.

Under low-P supply, SDW and PUE (calculated as the SDW produced per mg of phosphorous applied as fertilizer; Bryla and Koide, 1998) covariates with RDW, the concentration of Ca, Mg, S, Na, Mn, Zn in the leaves, and CKs (*t*-Z), ABA and Ca in the xylem sap along PC1, that explained 56.4% of the variability (**Figure 2**). In contrast, the shoot growth parameters were negatively associated with the concentration of the hormones SA, GAs and the ethylene precursor ACC. Most of nutrients in the xylem sap (P, K, Mn, Mg, Zn, Na, S), and the K, Fe, and P concentration in the leaves were clustered along PC2, explaining 43.6% of the variability, but orthogonal to the growth parameters. P concentration in both xylem and leaves was negatively associated to JA concentration and sap flow in the xylem (**Figure 2**). Other factors, rather than P concentrations, such as CKs, ACC, Ca, S, and some micronutrients seem to be more important in regulating shoot growth.

### Phosphorus and PUE

**FIGURE 3:**
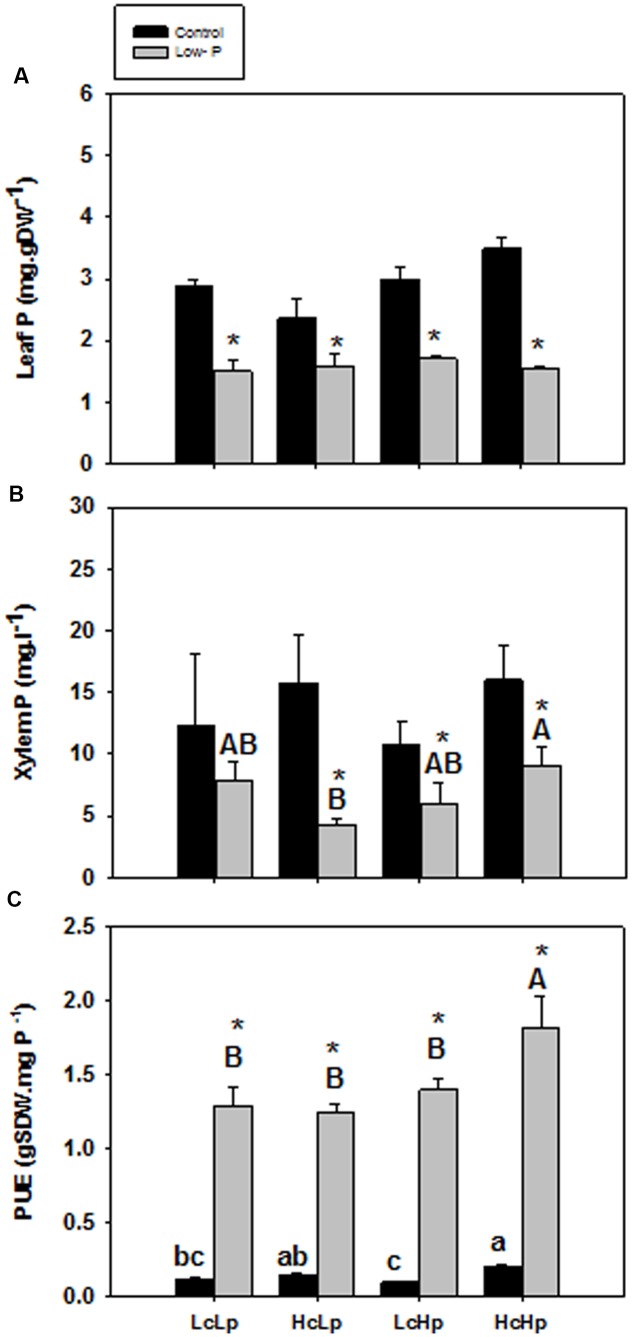
**Phosphorus concentration in leaf (A)** and xylem sap **(B)** and P use efficiency (PUE, calculated as the SDW produced per mg of phosphorus applied) **(C)** of the scion (*Solanum lycopersicum* cv. Boludo F1) grafted onto selected rootstocks for high (*H*) or low (*L*) vigor growing under control (*c*) and low P (*p*) conditions during 30 days. Different letters indicate significant differences among graft combinations (*n* = 6, *P* < 0.05) within each treatment. ^∗^indicate significant differences between control and low-P treated plants according to the Tukey test (*P* < 0.05).

**FIGURE 4:**
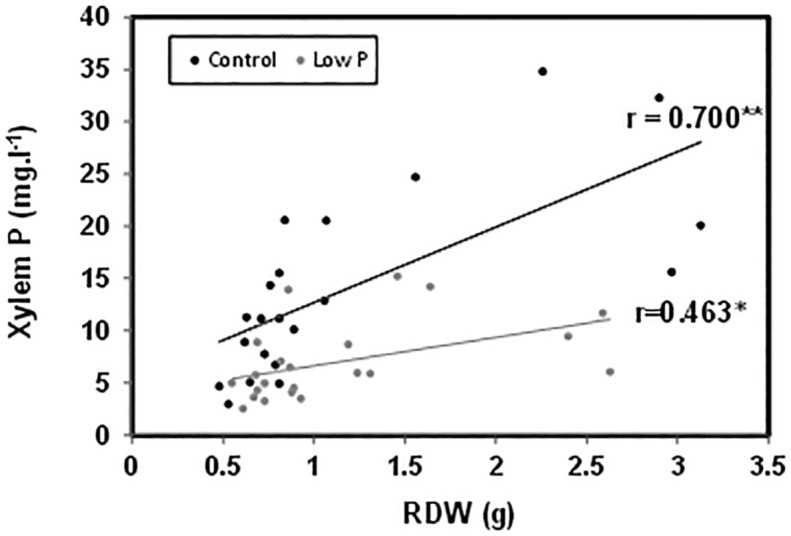
**Correlation between RDW and phosphorus concentration in the xylem sap of the scion (*Solanum lycopersicum* cv. Boludo F1) grafted onto selected rootstocks for high (*H*) or low (*L*) vigor growing under control (*c*) and low-P (*p*) conditions during 30 days. ^∗^*P* < 0.05; ^∗∗^*P* < 0.01, *n* = 22**.

P concentration in the root xylem sap and in the leaf was not significantly affected by the graft combination under normal P nutrition (**Figures 3A,B**). However, those concentrations were reduced between 30 and 50% in all plants under low-P. The sensitive *HcLp* rootstocks registered the greatest decrease (70% of control) and the lowest P concentration (<5 mg l^-1^, up to 50% lower than in the other plants) in the xylem sap, but reached similar concentration in the leaf (1.5 mg g^-1^ DW) than the other graft combinations (**Figures 3A,B**). Indeed, neither xylem or foliar P concentration was correlated with SDW under low-P supply (**Table 1** and **Figure 2**). However, P in the xylem was positively correlated with RDW under standard (*r* = 0.70, *P* ≤ 0.01) and low-P (*r* = 0.46, *P* ≤ 0.05) fertilization (**Figure 4**), supporting a general positive role for the root biomass in P uptake under whatever P nutrition.

PUE increased in all plants between 7 (*HcLp*) and 16 (*LcHp*) times under low-P compared to controls, and it was higher in the vigorous *HcHp* plants than in the other graft combinations under both growing conditions (**Figure 3C**). Curiously, the tolerant *LcHp* plants registered the lowest PUE under standard fertilization. An important question to address is why growth is stimulated in the tolerant *LcHp* grafted-plants that showed only slightly higher (non-significant) leaf P concentration (1.7 mg g^-1^ DW) than the sensitive *HcLp* ones?

### Calcium, Potassium, Magnesium, Sodium, and Sulfur

Ca concentration was only affected by the rootstock genotype under low-P supply but without clear relationship between xylem sap and the leaf (**Table 2**). Ca in the xylem was 2-fold higher in the vigorous *HcHp* plants than in the others, while the highest concentration of this nutrient in the leaf under low-P was found in the tolerant *LcHp* grafts. Under low-P, leaf Ca concentration was only reduced in the low vigor *LcLp* plants (**Table 2**). This nutrient seems to have an important role in the rootstock-mediated vigor since Ca concentration in both xylem and leaves was positively correlated with SDW (*r* = 0.43, 0.51, *P* ≤ 0.05) under low-P, which was probably due to a more efficient uptake by a greater RDW (*r* = 0.43, *P* ≤ 0.05) (**Table 1**). Similarly, the S concentration in the leaves under low-P was higher in the *Hp* grafts, while a decrease was registered in the *Lp* plants compared to the controls (**Table 2**), as supported by the positive correlation with SDW (*r* = 0.67, *P* ≤ 0.05) (**Table 1**). There were no significant differences between rootstocks genotypes in either xylem or leaf Na concentration under P deprivation, however, a positive correlation was found between Na concentration and RDW (*r* = 0.61, *P* ≤ 0.05) (**Table 1**). Little or no effects were observed for K and Mg (**Table 2**).

**Table 2:** Calcium (Ca), potassium (K), magnesium (Mg), sodium (Na), sulfur (S), manganese (Mn), boron (B), iron (Fe), and zinc (Zn) concentrations, in leaf and root xylem sap of the scion (*Solanum lycopersicum* cv. Boludo F1) grafted onto selected rootstocks for high (H) or low (L) vigor growing under control and low P conditions during 30 days.

			CONTROL		LOW-P		

			**Leaf (mg.gDW^-1^)**	**Xylem (mg.l^-1^)**	**Leaf (mg.gDW^-1^)**	**Xylem (mg.l^-1^)**	
**Macronutrients**	**Ca**	LcLp	27.41 ± 0.39	34.28 ± 12.51	18.30 ± 1.24 cˆ*	40.68 ± 7.40 b	
		HcLp	22.68 ± 2.72	44.93 ± 10.85	21.61 ± 0.32 b	33.29 ± 5.91 b	
		LcHp	23.47 ± 0.15	28.87 ± 4.16	25.60 ± 0.51 a	24.48 ± 6.54 b	
		HcHp	22.98 ± 0.39	57.92 ± 14.68	23.53 ± 0.18 ab	69.96 ± 7.95 a	
	**K**	LcLp	30.08 ± 0.33	341.64 ± 74.73	25.50 ± 3.74	316.07 ± 31.59 a	
		HcLp	31.42 ± 1.54	350.26 ± 60.82	25.41 ± 2.85	240.24 ± 29.24 ab	
		LcHp	30.30 ± 4.32	289.67 ± 32.38	28.58 ± 0.29	214.18 ± 25.14 b	
		HcHp	34.49 ± 1.94	347.74 ± 55.06	27.47 ± 1.78	285.00 ± 28.19 ab	
	**Mg**	LcLp	11.98 ± 1.52	24.22 ± 7.52	7.73 ± 0.81	29.97 ± 5.27	
		HcLp	10.45 ± 1.75	39.23 ± 5.41	7.54 ± 0.65	31.17 ± 5.01	
		LcHp	10.60 ± 0.01	30.73 ± 6.61	10.33 ± 0.56	22.24 ± 3.84	
		HcHp	10.80 ± 0.37	32.31 ± 6.18	9.48 ± 0.05	32.65 ± 3.18	
	**Na**	LcLp	5.44 ± 0.30 a	33.82 ± 14.27	3.02 ± 0.31	28.96 ± 6.95	
		HcLp	3.78 ± 0.56 b	30.79 ± 4.70	4.03 ± 1.12	25.04 ± 4.28	
		LcHp	3.48 ± 0.57 b	27.23 ± 6.95	2.91 ± 0.32	21.08 ± 4.78	
		HcHp	3.86 ± 0.38 b	17.10 ± 0.52	3.80 ± 0.50	13.42 ± 1.51	
	**S**	LcLp	9.20 ± 0.35 ab	11.49 ± 4.07	5.55 ± 0.85 bˆ*	16.42 ± 3.61	
		HcLp	8.70 ± 0.67 b	19.80 ± 2.39	6.50 ± 0.11 abˆ*	14.21 ± 1.49	
		LcHp	9.32 ± 0.58 ab	18.08 ± 5.25	8.59 ± 1.15 a	9.73 ± 1.39	
		HcHp	10.97 ± 0.89 a	18.34 ± 5.35	8.96 ± 0.21 a	18.21 ± 3.73	
**Micronutrients**	**Mn**	LcLp	0.23 ± 0.05 a	0.36 ± 0.18	0.15 ± 0.01 b	0.50 ± 0.13	
		HcLp	0.12 ± 0.14 b	0.44 ± 0.05	0.13 ± 0.00 b	0.42 ± 0.09	
		LcHp	0.24 ± 0.03 a	0.39 ± 0.09	0.20 ± 0.00 a	0.25 ± 0.06	
		HcHp	0.19 ± 0.02 ab	0.43 ± 0.08	0.18 ± 0.01 a	0.44 ± 0.05	
	**B**	LcLp	0.06 ± 0.01	Nd	0.04 ± 0.00	0.14 ± 0.01	
		HcLp	0.05 ± 0.01	Nd	0.05 ± 0.00	0.00 ± 0.00	
		LcHp	0.05 ± 0.00	Nd	0.04 ± 0.01	0.08 ± 0.01	
		HcHp	0.08 ± 0.01	0.06 ± 0.00	0.05 ± 0.00 ˆ*	0.03 ± 0.01	
	**Fe**	LcLp	0.13 ± 0.01	0.14 ± 0.06	0.09 ± 0.00b	0.18 ± 0.05	
		HcLp	0.12 ± 0.02	0.17 ± 0.05	0.12 ± 0.01a	0.11 ± 0.03	
		LcHp	0.18 ± 0.03	0.19 ± 0.05	0.13 ± 0.01a	0.10 ± 0.04	
		HcHp	0.17 ± 0.03	0.57 ± 0.34	0.11 ± 0.01b	0.20 ± 0.04	
	**Zn**	LcLp	0.07 ± 0.01	0.55 ± 0.36	0.03 ± 0.00ˆ*	0.47 ± 0.12	
		HcLp	0.04 ± 0.14	0.48 ± 0.14	0.05 ± 0.01	0.38 ± 0.13	
		LcHp	0.06 ± 0.01	0.41 ± 0.13	0.04 ± 0.01	0.27 ± 0.11	
		HcHp	0.06 ± 0.01	0.50 ± 0.16	0.06 ± 0.01	0.39 ± 0.12	

*Different letters indicate significant differences among graft combinations (*n* = 6, *P* < 0.05) within each treatment. ^∗^indicate significant differences between control and low P treated plants according to the Tukey test (*P* < 0.05). Nd = Not detected*.

### Micronutrients

Rootstock genotype and P nutrition had no significant effects on micronutrients B, Fe, and Zn. Only leaf Mn concentration was correlated with SDW under low P conditions (**Table 1**), and *Hp* rootstocks had greater leaf Mn concentrations compared to *Lp* rootstocks (**Table 2**).

### Hormone Concentrations in Root Xylem Sap

#### Cytokinins

**FIGURE 5:**
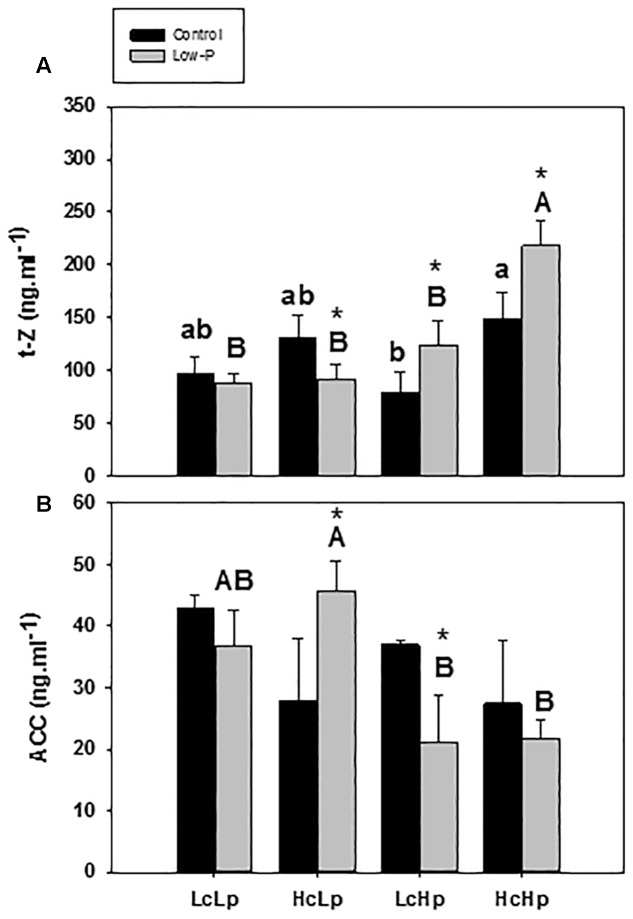
***Trans*-zeatin, *t*-Z (A)** and ACC **(B)** concentrations in root xylem sap of the scion (*Solanum lycopersicum* cv. Boludo F1) grafted onto selected rootstocks for high (*H*) or low (*L*) vigor growing under control (*c*) and low-P (*p*) conditions during 30 days. Different letters indicate significant differences among graft combinations (*n* = 6, *P* < 0.05) within each treatment. ^∗^indicate significant differences between control and low-P treated plants according to the Tukey test (*P* < 0.05).

The zeatin-type CKs (*t*-Z) were more abundant than the iP-type CKs in the root xylem sap (**Figure 5A** and **Table 3**). Zeatin concentration was 30–50% higher in the *Hc* rootstocks than in the *Lc* ones (**Figure 5A**). Low-P provoked a 30% increase in *t-Z* in the vigorous *Hp* rootstocks while no effect or reduction was observed in the low vigor (*LcLp*) and sensitive (*HcLp*) ones, respectively. ZR was also reduced in the sensitive *HcLp* plants under low-P, while it was unaffected in the tolerant *Hp* rootstocks (**Table 3**). No effects of the genotype or the treatment were observed on iP, while total CKs analyzed (Z+ZR+iP) in the xylem sap decreased in the sensitive *HcLp* and increased in the tolerant *LcHp* under low-P, while the concentration was 2-fold higher in the vigorous *HcHp* rootstocks under P deprivation than in the other graft combinations (**Table 3**). Indeed, *t-Z* and total CKs were positively correlated with shoot biomass under low-P conditions (*r* = 0.5, *P* ≤ 0.05), while the iP was negatively correlated with RDW (*r* = –0.59, *P* ≤ 0.01) (**Table 1**).

**Table 3:** Concentrations (ng ml^-1^) of zeatin riboside (ZR), isopentenyladenine (iP), total cytokinins (CKs), abscisic acid (ABA), jasmonic acid (JA), salicylic acid (SA), gibberellin A1 (GA_1_), gibberellin A3 (GA_3_), gibberellin A4 (GA_4_) and total gibberellins (GAs) concentrations in root xylem sap of the scion (*Solanum lycopersicum* cv. Boludo F1) grafted onto selected rootstocks for high (*H*) or low (*L*) vigor growing under control (*c*) and low P (p) conditions during 30 days.

	ZR	iP	CKs	ABA	JA	SA	GA_1_	GA_3_	GA_4_	GAs

**CONTROL**								
**LcLp**	26.05 ± 9.48	6.62 ± 0.07	109.13 ± 22.51	7.76 ± 1.12	12.38 ± 3.75	18.64 ± 5.80	5.05 ± 1.23	6.36 ± 0.10 a	6.39 ± 0.13	9.74 ± 3.45
**HcLp**	28.06 ± 8.07	4.68 ± 1.15	148.60 ± 24.16	10.20 ± 1.67	15.29 ± 2.94	21.58 ± 5.90	4.67 ± 1.01	6.22 ± 0.01 a	6.38 ± 0.07	13.06 ± 3.66
**LcHp**	12.95 ± 1.91	3.99 ± 0.96	89.79 ± 19.32	14.37 ± 4.60	10.70 ± 1.79	30.05 ± 10.93	3.64 ± 0.91	3.88 ± 0.97 b	4.32 ± 1.06	10.97 ± 2.94
**HcHp**	21.09 ± 7.56	4.30 ± 1.35	162.19 ± 24.16	10.16 ± 0.93	11.08 ± 1.97	12.77 ± 0.68	4.34 ± 0.95	5.62 ± 0.65 a	4.09 ± 1.25	9.88 ± 2.91

**LOW-P**								

**LcLp**	15.78 ± 0.00	6.36 ± 0.70	96.36 ± 10.24 b	9.59 ± 1.20	11.37 ± 2.64	25.63 ± 9.95	5.08 ± 0.88	6.22 ± 0.07	6.41 ± 0.11	12.46 ± 3.27
**HcLp**	Nd	6.12 ± 0.61	97.11 ± 14.08 b	8.64 ± 1.01	13.90 ± 1.84	17.16 ± 1.71	5.95 ± 0.45	5.91 ± 0.53	4.76 ± 1.01	13.06 ± 2.53
**LcHp**	12.47 ± 3.23	5.36 ± 1.24	133.13 ± 21.87 b	7.58 ± 0.35	14.53 ± 1.18	16.83 ± 2.04	5.77 ± 0.54	5.37 ± 0.78	5.78 ± 0.64	11.30 ± 3.04
**HcHp**	26.29 ± 9.41	5.98 ± 0.58	231.76 ± 27.2 a	11.14 ± 1.81	10.09 ± 1.31	12.12 ± 1.53	5.85 ± 0.43	6.24 ± 0.10	5.10 ± 0.85	13.29 ± 2.84

*Nd = Not detected. Different letters indicate significant differences among graft combinations (*n* = 6, *P* < 0.05) within each treatment*.

### 1-Aminocyclopropane-1-Carboxylic Acid

Under standard P nutrition, ACC concentration was higher in the low vigor (*Lc*) than in the high vigor (*Hc*) rootstocks, but the differences were not significant (**Figure 5B**). Low-P supply increased ACC concentration in the sensitive *HcLp* plants while it decreased in the tolerant *LcHp* ones. The highest and lowest concentrations were registered in the low (*Lp*) and high (*Hp*) vigor rootstocks, respectively, existing a negative correlation with SDW and RDW (*r* = –0.49, *P* ≤ 0.05). Therefore, this negative relationship between ACC in the xylem and shoot performance suggests that ACC is a main player in the constitutive rootstock-mediated vigor and in the adaptive shoot growth regulation under low-P.

#### Gibberellins and Abscisic, Salicylic, and Jasmonic Acids

No significant differences in GAs, ABA, SA, and JA were observed between genotypes or treatments (**Table 3**).

## Discussion

### Tomato Rootstocks Can Improve Shoot Growth and PUE under Low-P Nutrition

Different tomato rootstocks generated a 1.5-fold variation in shoot biomass of a unique commercial scion variety under low-P nutrition (Albacete et al., 2015b), exemplified by the low (*LcLp*) and high (*HcHp*) vigor rootstocks (**Figure 1A**) that improved PUE (**Figure 3C**). This variation was partially due to general rootstock-mediated vigor, since shoot biomass was significantly positively correlated under control and low-P conditions (data not shown), as in the low (*LcLp*) and high (*HcHp*) vigor lines. Interestingly, in addition to low and high vigor lines where vegetative growth was insensitive to low-P, other rootstocks had a normal sensitive growth reduction in response to low P (*HcLp*), while others doubled their shoot biomass under low-P conditions, when compared to controls (*LcHp*). Genetically, the rootstocks conferring low-vigor (*LcLp*) and sensitive (*HcLp*) traits belong to the RIL population from *S. lycopersicum × S. pimpinellifollium*. Rootstocks conferring high-vigor (*HcHp*) belong to *S. lycopersicum* var. cerasiforme, and were previously chosen for whole plant drought tolerance (as whole plants), and as rootstocks promoting vigor (top 7% of the population) under high soil impedance, low-K supply, and also under control conditions (Albacete et al., 2015a,b). The two rootstocks with positive growth response under low-P (*LcHp*) were also selected for salinity (IL from *S. lycopersicum × S. pennellii*) and drought (RIL 233) tolerance (data not shown). Therefore, while high or low induced vigor is likely due to common general physiological processes that affect scion growth, as in vigorous commercial F1 rootstocks Maxifort and Beaufort (derived from *S. lycopersicum × S. habrochaites*, data not shown), the negative or positive growth response under low-P may be due to more stress-specific mechanisms.

### Changes in Root Growth Do Not Influence P Uptake Nor Shoot Performance under Low-P Supply

Although root biomass was correlated with xylem P concentration (**Figure 4**), differences in rootstock-mediated shoot vigor under low-P were not explained by differences in root growth, since all graft combinations had a similar root biomass (**Figure 1A**) and leaf P concentration (**Figure 3B**). While P starvation is expected to increase root/shoot ratio (Nagarajan and Smith, 2012), no effects were detected (**Figure 1C**) perhaps due to limited treatment duration (30 days). Indeed, the lack of root growth response may be because a threshold leaf P concentration (<0.13%) was not reached (Besford, 1979) despite the strong decrease (up to 70%) in P uptake and transport in the xylem (**Figures 3A,B**). Moreover, since the roots were constrained to a limited (2 l) volume during the experiment and nutrient solution was added regularly, it is unlikely that differences in RSA were responsible for different shoot growth responses and P uptake.

Similarly, the capacity to absorb and transport P under low-P can only explain the shoot growth sensitivity of the *HcLp* rootstocks, which decreased by 70% compared to control conditions, while this reduction was around 40% in the other 3 graft combinations (**Figure 3B**). However, foliar P concentration was similar in all graft combinations under low-P and 30% (*HcLp*) to 50% (*HcHp*) lower than under control conditions (**Figure 3A**) and only the shoot growth of the sensitive graft-combination *HcLp* was limited by the reduced P uptake, as reflected in the lowest PUE (30% lower than the highly efficient *HcHp* rootstock). These results suggest that below a threshold P concentration in the xylem sap, the shoot growth is reduced in order to reach a growth-compatible leaf P concentration. Therefore, shoot growth may adjust to low xylem P delivery, rather than the leaf P concentration, since a weak positive correlation (*r* = 0.23, *P* ≤ 0.01) was found between xylem P concentration and SDW under low-P in the whole population (data not shown). Although low-P can decrease root hydraulic conductivity (Radin, 1990), the different graft combinations showed no significant differences in root biomass or sap flow (**Figures 1B,D**). Therefore, although root biomass influences root P export (**Figure 4**), adaptive changes in root growth and/or P uptake cannot explain the growth phenotypes observed under low-P supply. The fact that rootstock-mediated shoot biomass is hardly explained by the capacity of the rootstock to uptake, transport and accumulate the limiting nutrient (P) to the aerial part of the plant is also supported by the PCA analysis.

### Rootstock-mediated Ca, S, and Mn Nutrition is Related to Shoot Performance under Low-P Supply

Besides P, xylem Ca concentration was also correlated with SDW and RDW under both high and low-P conditions (**Table 1**). Constitutive rootstock capacities to uptake Ca and to maintain high foliar Ca concentrations seem related to the general vigor (*HcHp*) and adaptability to low-P (*LcHp*), as under low-K conditions (Martínez-Andújar et al., 2016). The positive role of Ca in adaptation to low-P was supported by the consistent correlation between xylem Ca and P concentrations (*r* = 0.6, *P* ≤ 0.01), and with RDW under low-P, suggesting that root biomass significantly influences the uptake of those nutrients. The positive relation between xylem P and Ca and RDW was significant for most of nutrients in the xylem sap under standard fertilization (data not shown), also supporting the expected relationship between root and shoot vigor through improved nutrient supply to the shoot, at least under control conditions. However, only xylem and leaf Ca concentrations were significantly correlated with SDW in the selected lines under low-P (**Table 1**).

Similarly to Ca, rootstock-mediated capacities to maintain high leaf S (*r* = 0.67, *P* ≤ 0.01) and Mn (*r* = 0.81, *P* ≤ 0.01) concentration were related to the shoot biomass produced under low-P (**Table 1**). Therefore, it seems that the rootstock-mediated shoot growth improvement under low-K or -P supply is related to the capacity to maintain high nutritional status for other nutrients such as Ca, S, and Mn, while the role of some micronutrients such as B and Zn seems more specific to positive rootstock effects under low-K (Martínez-Andújar et al., 2016). The physiological role of those nutrients in adapting to low-P could be explained by hormonal or metabolic interactions, as discussed below.

### The Ethylene-precursor ACC (Negative) and *Trans*-zeatin (Positive) Are Root-to-Shoot Signals Regulating Shoot Growth under Low-P

In addition to rootstock constitutive nutrient uptake and export capacity, low-P can alter hormone transport to the shoot to influence scion growth and adaptive responses. Currently, ACC and cytokinins have been proposed to be xylem-mediated root-to-shoot signals in response to P deficiency (Franco-Zorrilla et al., 2005; Lough and Lucas, 2006; Lucas et al., 2013; Lin et al., 2014; Zhang et al., 2014; Song et al., 2015), while JA is receiving growing attention (Khan et al., 2016).

P deficiency alters root ethylene biosynthesis (Borch et al., 1999; Lynch and Brown, 2001; Li et al., 2009; Song et al., 2015) and responsiveness (He et al., 1992; Kim et al., 2008), but without a clear pattern. Following P deprivation, ethylene emission from maize and tomato roots decreased (Drew et al., 1989; Kim et al., 2008), while it increased from roots of common bean (*Phaseolus vulgaris*), white lupin (*Lupinus albus*), and *Medicago falcata* (Borch et al., 1999; Gilbert et al., 2000; Li et al., 2009). Enhanced expression of ethylene biosynthetic genes (e.g., ACC oxidase, ACC synthase) has also been involved in shoot P responses (Misson et al., 2005; Kim et al., 2008). Whether increased shoot ethylene biosynthesis is regulated by shoot P levels or by root-sourced signals is not known (Song et al., 2015), but it seems that ethylene can also be transported from the roots in the form of its precursor ACC (Else and Jackson, 1998; Albacete et al., 2008). In this study (**Figure 5B**), the three responses (increase, decrease or no change in xylem ACC concentration) occurred in the same species depending on the root genotype and the relation found with the growth response of the scion suggests a role for ACC in the systemic signaling under low-P. In this regard, the induction of ethylene precursor ACC in the xylem of sensitive *HcLp* graft combination could be the putative root-to-shoot signal inhibiting shoot growth in response to the low P uptake (only 30% of control). However, the ACC response was not observed in the insensitive *LcLp* and *HcHp* rootstocks, which maintained growth despite 40% lower xylem P concentrations, although the ACC concentrations were inversely related to the transmitted vigor. Interestingly, the ACC levels were depressed in the tolerant *LcHp* rootstocks, which showed increased growth under low-P. These ACC responses in contrasting rootstocks under low-P were similar to those observed under low-K (Martínez-Andújar et al., 2016), and under non-stress conditions in hydroponics (Cantero-Navarro et al., 2016), suggesting that ethylene-precursor is a constitutive or stress-induced root-to-shoot negative signal regulating shoot growth.

According to the PCA (**Figure 2**), several nutritional and hormonal parameters were positive and negatively associated to SDW and xylem ACC concentration, respectively, suggesting that ethylene could influence shoot growth directly and/or regulating the putative negative ACC signal coming from the roots. This regulation could be via signal concentration and/or perception (sensitivity). Leaf assimilation of some nutrients such as Ca, S, and Mn was significantly higher in the high vigor *Hp* rootstocks than in the low vigor *Lp* ones under low-P supply and were negatively correlated with xylem ACC concentration (*r* = –0.81 to –0.92, *P* ≤ 0.05, data not shown). Increased foliar S, Ca, and Mn concentrations may not only attenuate a putative feedback signal to the roots thus reducing the ACC signal in the xylem, but also decreases sensitivity to ethylene in the shoot in the *Hp* graft combinations. Indeed, S nutrition has been reported to modulate the stress response by altering ethylene production and signaling in several stresses, although the mechanisms are still obscure (Wawrzynska et al., 2015). Experiments in mustard and wheat indicated that additional S supply reduced sensitivity to ethylene under Cd toxicity, probably by alleviating oxidative stress (Khan et al., 2015). Replacement of phospholipids by sulfolipids in membranes has also been reported as a metabolic adaptation to P-impoverished soils in Proteaceae (Lambers et al., 2011). The role of those nutrients in the rootstock-mediated adaptation to low-P deserves further investigation.

Among the other hormones studied, the bioactive cytokinin t-Z decreased in the xylem sap of the sensitive *HcLp*, increasing in the tolerant *LcHp* and with inverse relationship to vigor induced by the low-P insensitive *LcLp* and *HcHp* rootstocks (**Figure 5**). Indeed, this hormone was correlated with the rootstock-mediated growth under both control and low-P conditions (*r* = 0.51, *P* ≤ 0.05). Those results indicate that maintaining high root *t*-Z export sustains growth under P deprivation suggesting this hormone is important in mediating general plant vigor. However, in addition to general plant performance, this CK seems to affect rootstock response to low-P by its effects on scion vigor. Exogenous CKs increase shoot P concentration and dramatically represses various P starvation responses such as anthocyanin accumulation and the induction of the P starvation responsive genes, while this repression is impaired in CK-receptor mutants (Martín et al., 2000; Franco-Zorrilla et al., 2005; Wang et al., 2006; Shi and Rashotte, 2012). Conversely, CK levels are down- or up-regulated by low or high P availability in the shoots, respectively (Ei-D et al., 1979; Horgan and Wareing, 1980; Lan et al., 2006). According to the expected effects of low-P on hormonal levels, only the sensitive graft combination *HcLp* responds by increasing the concentration of the putative negative growth regulator ACC and decreasing cytokinins (*t*-Z) as positive growth regulator (Zhang et al., 2014). Therefore, CKs and ACC are considered to play an important role in root to shoot signaling under P starvation (Martín et al., 2000; Zhang et al., 2014) and may comprise part of a systemic feed-back signaling system adapting P availability to shoot growth, as proposed for tomato fruit growth under salinity (Albacete et al., 2014).

Finally, the inverse relationship between xylem JA and P concentrations and also foliar concentrations of these analytes (*r* = –0.82, *P* ≤ 0.01, **Figure 2**) indicates that this hormone may also act as a systemic root-to-shoot systemic signal in response to low-P, which deserves further attention (Morcuende et al., 2007; Khan et al., 2016).

## Conclusion

Rootstock-mediated regulation of scion growth under low-P depends on both constitutive and induced mechanisms that involve nutritional (P, Ca, S, and Mn) and hormonal (ethylene and cytokinins) traits. While rootstock-mediated vigor under non-stress conditions is generally related to all those factors, the growth reduction under low-P is only explained by decreased root P export rather than by low foliar P concentrations. Therefore, low P uptake could induce a negative (ACC) or reduce a positive (*t*-Z) signal to adapt scion vegetative growth to rootstock P export. The use of *Hp*-type rootstocks can convert a commercial scion variety into a P-efficient phenotype, which may increase yield stability in P deficient soils and/or allow lower P fertilizer applications.

## Author Contributions

CM-A, JR-L, and FP-A designed the research; JR-L, CM-A, and AA performed research, data collection and analysis; CM-A, JR-L, and FP-A performed data interpretation; JR-L, CM-A, ID, and FP-A performed critical revision of the article; CM-A and FP-A drafted the article; JR-L, ID, and FP-A carried out final approval of the final version.

## Conflict of Interest Statement

The authors declare that the research was conducted in the absence of any commercial or financial relationships that could be construed as a potential conflict of interest.
